# Fine-tuning of mechanical properties in a Zn–Ag–Mg alloy via cold plastic deformation process and post-deformation annealing

**DOI:** 10.1016/j.bioactmat.2021.03.017

**Published:** 2021-03-21

**Authors:** Maria Wątroba, Wiktor Bednarczyk, Jakub Kawałko, Piotr Bała

**Affiliations:** a AGH University of Science and Technology, Faculty of Metals Engineering and Industrial Computer Science, Al. A. Mickiewicza 30, 30-059 Krakow, Poland; b AGH University of Science and Technology, Academic Centre for Materials and Nanotechnology, Al. A. Mickiewicza 30, 30-059 Krakow, Poland

**Keywords:** Zinc alloys, Cold rolling, Heat treatment, Mechanical properties, Grain refinement

## Abstract

In recent years, Zn-based materials have been extensively investigated as potential candidates for biodegradable implant applications. The introduction of alloying elements providing solid-solution strengthening and second phase strengthening seems crucial to provide a suitable platform for the thermo-mechanical strengthening of Zn alloys. In this study, a systematic investigation of the microstructure, crystallographic texture, phase composition, and mechanical properties of a Zn–3Ag-0.5Mg (wt%) alloy processed through combined hot extrusion (HE) and cold rolling (CR), followed by short-time heat treatment (CR + HT) at 200 °C was conducted. Besides, the influence of different annealing temperatures on the microstructure and mechanical properties was studied. An adequate combination of processing conditions during CR and HT successfully addressed brittleness obtained in the high-strength HE Zn–3Ag-0.5Mg alloy. By controlling the microstructure, the most promising results were obtained in the sample subjected to 50% CR reduction and 5-min annealing, which were: ultimate tensile strength of 432 MPa, yield strength of 385 MPa, total elongation to failure of 34%, and Vickers microhardness of 125 HV0.3. The obtained properties clearly exceed the mechanical benchmarks for biodegradable implant materials. Based on the conducted investigation, brittle multi-phase Zn alloys' mechanical performance can be substantially enhanced to provide sufficient plasticity by grain refinement through cold deformation process, followed by short-time annealing to restore proper strength.

## Highlights

• Multiphase Zn–3Ag-0.5 Mg alloy exhibits high strength and limited plasticity.
• Cold rolling (CR) eliminates the brittleness obtained in the hot-extruded state.
• Grain refinement and second phases' fraction control the plasticity in the CR state.
• Short-time heat treatment restores the strength reduced after CR.
• The best obtained mechanical results are UTS of 432 MPa, YS of 322 MPa, E_F_ of 34%.

## Graphical abstract

**Figure:**
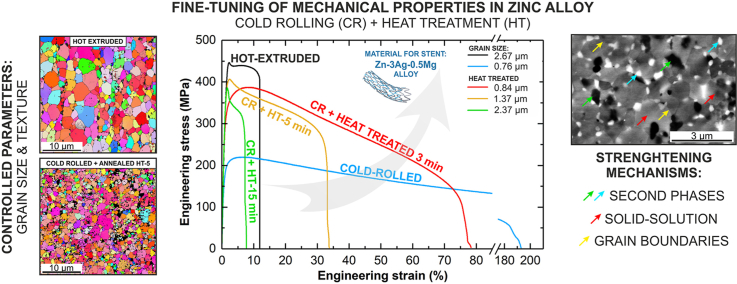

## 1 Introduction

Zn and its alloys have gained increasing attention as biodegradable metallic materials due to the acceptable biocompatibility and moderate corrosion rate [1]. Some concerns have arisen lately that Zn alloys may exhibit a cytotoxic effect when tested with different cell lines depending on the alloying elements selection. Nevertheless, it has been already found that *in vitro* experiments conducted in accordance with standards typically used for non-degradable materials are not always suitable to predict the biocompatibility of Zn alloys. The cytotoxic effect is rarely directly triggered by the alloying element, especially at their low concentrations, but is rather caused by Zn alloys' degradation behavior in used culture media and the amount of released Zn^2+^ ions that affect the cell viability [2,3]. As a result, despite the *in vitro* experimental results indicating a cytotoxic effect of Zn alloys, there are studies confirming their excellent *in vivo* biocompatibility, no harmful effects on the animal organs, good hemocompatibility, proper vascular healing, or new bones formation [[4], [5], [6], [7]].

Apart from biological properties, the potential for the use of pure Zn as a biodegradable implant material is limited by its insufficient mechanical properties, including low strength and lack of adequate plasticity. Zn's mechanical properties also suffer from other aspects related to recrystallization at low temperature, high strain rate sensitivity at room temperature (RT), and possible natural aging or creep at RT [8].

Extensive research is aimed at designing a suitable chemical composition of Zn alloys that would address all of the above-mentioned mechanical issues. The introduction of alloying elements results in grain refinement, solid-solution strengthening [9], second-phase hardening [10], but at the same time should not affect the optimal corrosion behavior and biocompatibility of Zn [4]. The grain refinement of a large, columnar, dendritic microstructure obtained in an as-cast state, provided by plastic deformation processes, is crucial since uniform and equiaxed refined grains result in desirable isotropic mechanical properties required in load-bearing medical applications. Grain refinement in metals typically delivers an improvement of strength and ductility. However, as shown recently in the literature, reducing the grain size in Zn alloys below a few micrometers can bring opposite results. For instance, the ultrafine-grained microstructure obtained via cold severe plastic deformation (SPD) method in low-alloyed Zn-0.5(Ag, Cu Mn) alloys resulted in an evident decrease in strength compared to the hot-extruded state (by nearly 50%) and activated superplasticity at RT [11,12]. Seeing that solid-solution strengthening is usually not sufficient to enable obtaining high-strength, ductile Zn alloy fulfilling the mechanical requirements for biodegradable implants (ultimate tensile strength UTS > 300 MPa, yield strength YS > 200 MPa, and elongation to failure ε_F_ > 18%) it is essential to provide second phase strengthening via alloying and proper thermomechanical processing conditions.

Firstly, the role of precipitates is to hinder the grain growth after dynamic recrystallization (DRX) observed during plastic deformation processes and strengthen the grain boundaries to hinder grain boundary sliding (GBS) responsible for superplasticity and strain softening [13,14]. Secondly, complex plastic deformation, including cold metalworking and further heat treatment (HT), should provide suitable Zn-based matrix grain size and texture, the formation of secondary precipitates, and the appropriate amount of low- (LAGB) and high-angle grain boundaries (HAGB) [[15], [16], [17]].

As reported, Zn alloys containing Mg additions exhibit satisfactory mechanical strength due to the presence of a highly strengthening Mg-rich eutectic phase [18,19]. However, the highly-soluble Ag addition can provide both solid-solution strengthening and improvement of mechanical properties by tuning the fraction of Ag-rich phases [5,20]. The current study aims to tailor both strength and ductility in the Zn–3Ag-0.5Mg alloy developed recently [21] to obtain properties significantly exceeding the mechanical requirements for biodegradable implant material using simple processing routes. The novelty in this study is the employment of a hot extrusion (HE) process directly after casting without inter-process annealing (used in our previous study). In addition, the influence of different thickness reductions during cold rolling (CR), the duration and temperature of short-time heat treatment (HT) on the microstructure evolution and mechanical properties of the Zn alloy was studied for the first time and will benefit the fabrication of biodegradable high-strength multi-phase Zn alloys.

## 2 Materials and methods

The material used in the present investigation is a Zn–3Ag-0.5Mg alloy (wt%). The alloy was prepared by melting commercially pure Zn (99.995 wt%), Ag (99.995 wt%), and Mg (99.95 wt%) at 650 °C for 30 min in a graphite crucible using an induction furnace, followed by gravity casting into a cold steel mold. The ingots weighing about 250 g were indirectly hot extruded at 250 °C with an extrusion ratio equal to 25. Next, the rods were subjected to multistep cold rolling at a constant rolling speed of 0.06 m/s in a laboratory rolling mill to impart four different thickness reductions: 30%, 50%, 75%, 90%. The final step was short-time post deformation annealing at 200 °C for 3, 5, and 15 min. The annealing temperature was selected to be lower than the extrusion temperature and higher than the typical sterilization temperature of biomedical devices (120 °C). Besides, short-time heat treatment was conducted on the CR90% sample at the temperature range of 50 °C ÷ 250 °C for 5 min to investigate the effect of annealing temperature on the microstructure and mechanical properties. The hot-extruded and cold-rolled alloys will be further referred to as HE, CR30%, CR50%, CR75%, CR90%, and for the heat-treated state by + HT-3, HT-5, and HT-15, and + HT-5 50 ÷ 250 depending on the annealing temperature.

Samples for microstructural observations were prepared via standard metallographic preparation methods using water-free diamond suspensions followed by low-angle Ar^+^ ion milling with Hitachi IM4000Plus Ion Milling System. The microstructure was analyzed using an FEI VERSA 3D scanning electron microscope (SEM) equipped with an electron backscattered diffraction (EBSD) detector. During EBSD data collection, the step size was set at least ten times smaller than the observed grain size. For the EBSD analysis, only the η-Zn hexagonally closed-packed (HCP) phase was indexed, whereas precipitates of second phases were excluded from the EBSD indexing. The elemental distribution map was collected via energy dispersive x-ray spectrometry (EDS) in an SEM using an EDAX microanalysis system. The approximate composition of the particular phases was determined based on spectra collected at 10 keV and analyzed by EDAX Team software utilizing ZAF correction and averaging several selected areas. Additionally, the phase composition was examined using a Panalytical Empyrean X-ray diffractometer with CuK_α_ radiation (α = 1.5406 Å). Diffractograms were collected in the 15 to 90° range of 2θ angles at 40 kV, 40 mA, with a scanning rate of 0.4°/min and a step size of 0.02°.

Uniaxial tensile tests were performed at a constant strain rate of 10^−3^ s^−1^ at RT using INSTRON 5966 universal testing machine. The mechanical properties were averaged from the measurements performed on three specimens. The average Vickers microhardness was calculated based on six measurements recorded using TUKON 2500 hardness tester under a 2.95 N (HV0.3) load with a dwell time of 10 s.

## 3 Results

### 3.1 Microstructure observations

**Fig. 1:**
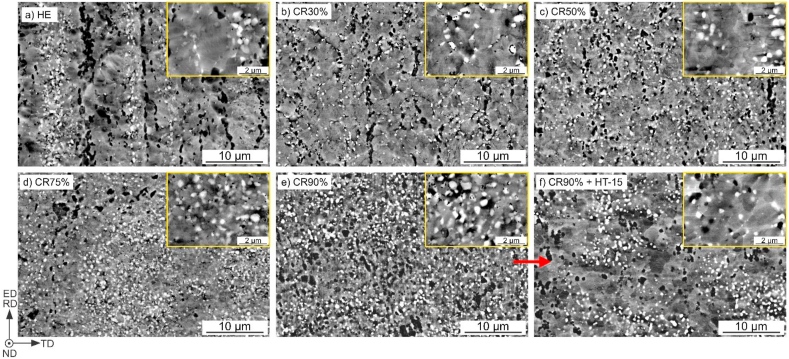
SEM-BSE images of samples after HE (a), CR (b–e), and CR90% followed by 15 min annealing (f) in the longitudinal cross-section.

**Fig. 2:**
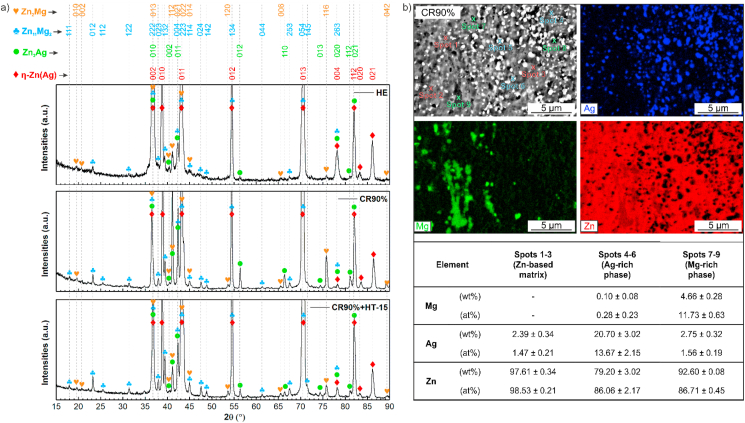
X-ray diffractograms of the HE, CR90%, and CR90% + HT-15 samples (a); and EDS elemental maps (b) with concentrations of elements measured in the selected points.

The microstructure of the Zn–3Ag-0.5Mg alloy in the as-cast state was characterized in detail in our previous work [21]. The material is composed of η-Zn(Ag) matrix dendrites and characteristic lamellar Mg-rich eutectic mixture in the interdendritic region after casting. Fig. 1 presents the microstructures of the Zn–3Ag-0.5Mg alloy in the HE (Fig. 1a), CR (Fig. 1b–e), and additionally CR90% + HT-15 states (Fig. 1f). The simultaneous addition of Ag and Mg resulted in multi-phase composition with precipitates present in the whole volume of material. According to detailed microscopic observations, including EDS analysis (Fig. 2b) and X-ray diffraction (XRD) measurements (Fig. 2a), the microstructure is composed of the η-Zn phase enriched in Ag addition, ε-Zn_3_Ag intermetallic phase in the form of white, small precipitates located both inside the grains and at grain boundaries (visible in Fig. 1 at higher magnifications). The darkest areas correspond to the grains of Mg-rich second phases. According to our previous study [21] on the same alloy system, these grains contain a mixture of nano-sized Zn_11_Mg and Zn_2_Mg phases enriched in Ag addition. These phases were identified on XRD diffractograms collected for HE, CR, and CR + HT samples. No differences between the phase compositions were observed in the examined samples, so the diffractograms for HE, CR90%, CR90% + HT-15 samples were selected to show in Fig. 2a.

**Fig. 3:**
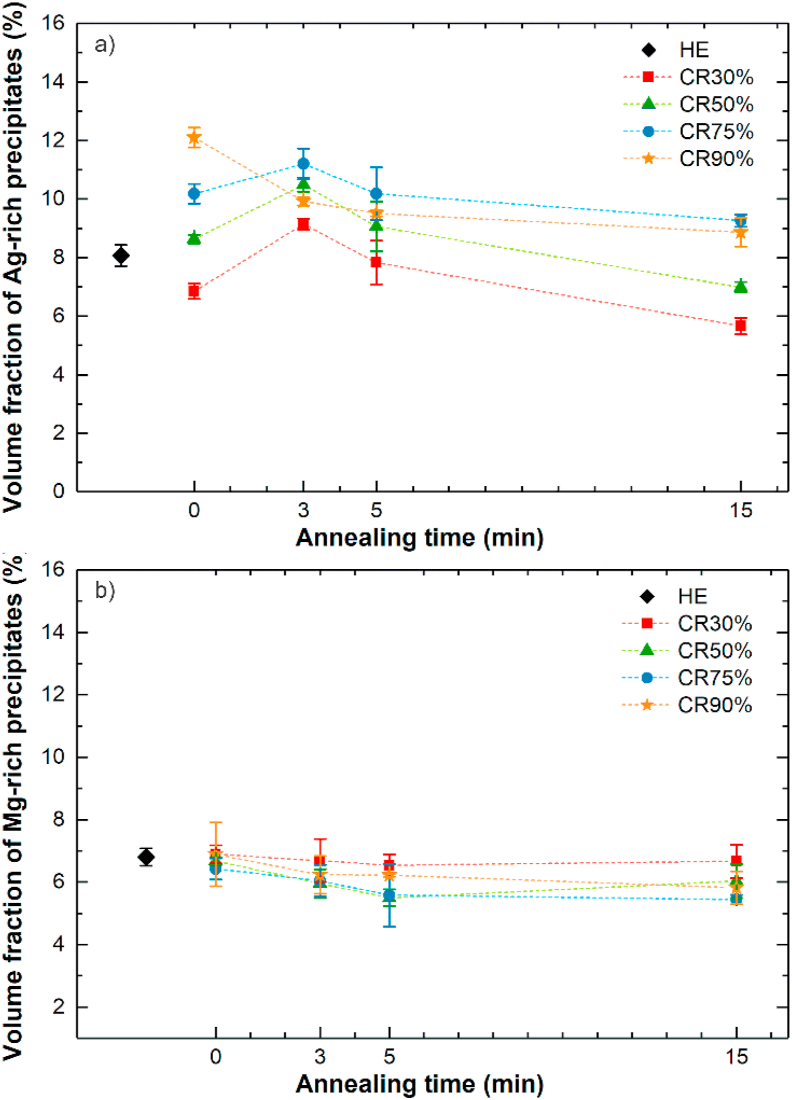
Variation of Ag-rich (a) and Mg-rich (b) precipitates' volume fractions vs. the applied annealing time. The results present the estimated mean value of volume fractions with standard deviation.

Microstructural analysis performed on longitudinal cross-sections in HE and CR states revealed bands consisting of precipitates distributed along the extrusion (ED) and rolling direction (RD). Based on SEM microstructural observations, the volume fraction of Ag-rich and Mg-rich phases were estimated and summarized in Fig. 3. As presented, growing CR deformation resulted in an increasing volume fraction of ε-Zn_3_Ag precipitates. Compared to the HE state lower amount of ε-Zn_3_Ag precipitates was determined only in the CR30% sample.

Seeing that cold deformation results in significant grain refinement and high density of HAGB being an easy diffusion path for Ag atoms, the second phase's precipitation from the supersaturated solid solution is more likely to occur rather than its dissolution, especially as a result of the applied strain [22]. Therefore, the abnormal decrease is probably connected with the overestimated Ag-rich second phase's volume fraction determined for the HE state. It can be affected by the local inhomogeneity related to the bands of precipitates visible in the microstructure (Fig. 1a) or measurement uncertainty accompanying SEM image analysis. The subsequent short-time HT resulted in general at first (after HT-3) in further increase in ε-Zn_3_Ag precipitates volume fraction and next in a continuous decrease associated with second phase dissolution with an increase in annealing time in all CR samples. According to the Zn–Ag phase diagram, the solubility of Ag in Zn changes with temperature, and the estimated maximum solubility at 200 °C is ~2.5 wt% of Ag and at RT ~0.3 wt%, so annealing the Zn–3Ag-0.5Mg alloy at 200 °C provides conditions close to the ε-Zn_3_Ag phase solvus line in the phase diagram. Consequently, Ag-rich particles' dissolution occurs to an extent depending on the annealing time. After annealing, the relatively fast cooling rate provides the enrichment of η-Zn(Ag) grains in Ag and leads to a decrease in ε-Zn_3_Ag phase volume fraction.

Microscopic analysis of the Mg-rich grains volume fraction indicates only small differences between the investigated samples, both after plastic deformation and heat treatment (Fig. 3b). The estimated volume fraction was in the range of 6.3 ± 0.4%, without a significant trend with respect to the applied cold work and annealing time. The observed differences in Mg-rich grains volume fraction may also be affected by measurement uncertainty resulting from SEM image analysis. Most importantly, based on the Zn–Mg phase diagram, Mg is almost insoluble in Zn, so the Mg-rich phases should be stable at 200 °C, and no actual changes in their volume fraction were expected [23].

### 3.2 SEM-EBSD measurements

**Fig. 4:**
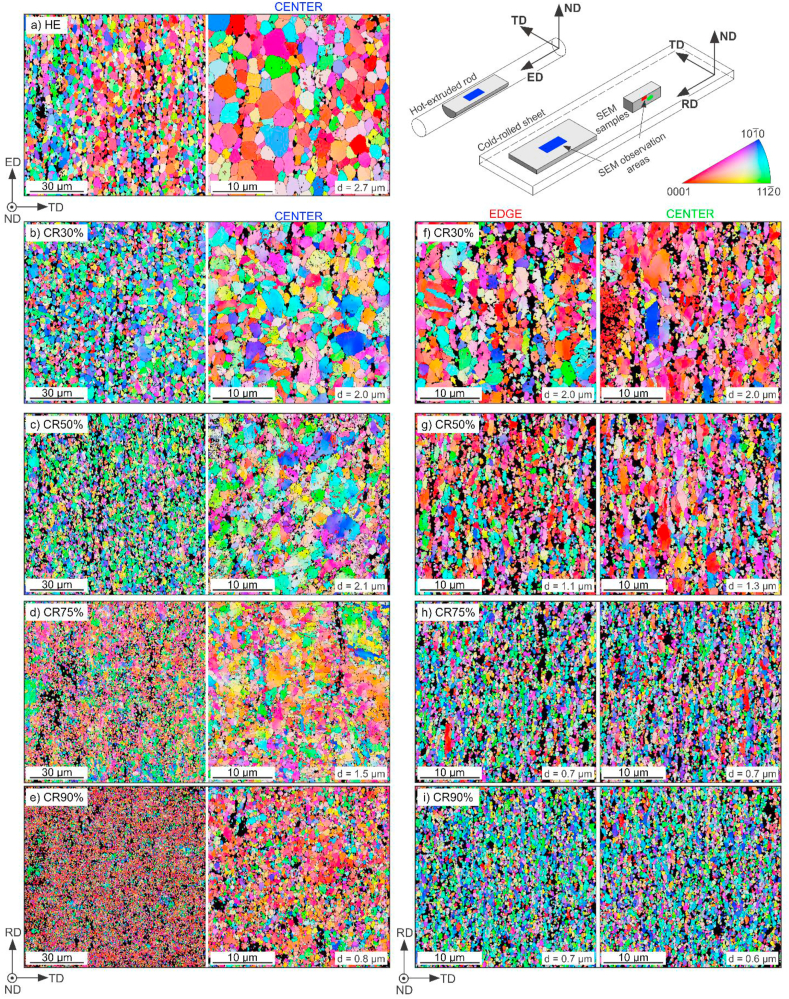
EBSD-IPF maps of Zn–3Ag-0.5Mg alloy after HE (a) collected in the longitudinal cross-section along the extrusion direction, and CR (b–e) collected in the rolling plane from the 100 × 100 μm (left) and 30 × 30 μm (right) areas. EBSD-IPF maps after CR collected from longitudinal cross-section (f–i). IPF maps are colored according to the normal direction of the samples' surface.

In Fig. 4, the Inverse Pole Figure (IPF) maps for HE (Fig. 4a), and CR samples in two perpendicular cross-sections were collected (Fig. 4b–i). Additionally, to show any possible changes in CR samples' microstructure, the IPF maps were collected in longitudinal cross-section in two areas in the thickness direction; first close to the edge of the sample and second in its center (Fig. 4f–i). Due to applied cold deformation and susceptibility of Zn to dynamic recrystallization at RT, the homogeneous microstructure in longitudinal cross-section with negligible differences in the thickness direction was observed in all CR samples. Only the slight anisotropy after the CR process seen as grains elongated in the rolling direction is visible and results in smaller average grain size than measured in the rolling plane. Because the gradual grain refinement with increasing CR total strain is maintained in both perpendicular directions, all EBSD data presented and analyzed further in the manuscript originate from the IPF maps collected in the rolling plane presented in Fig. 4b–e. IPF maps after HE and CR were also collected from the 100 × 100 μm area to show the deformed microstructure's evolution (Fig. 4a–e, left column). Second phases' precipitates (mainly Mg-rich grains) are visible as black spots arranged in bands along with the ED and RD. However, the bands vanish with a higher CR reduction and become more randomly distributed.

**Fig. 5:**
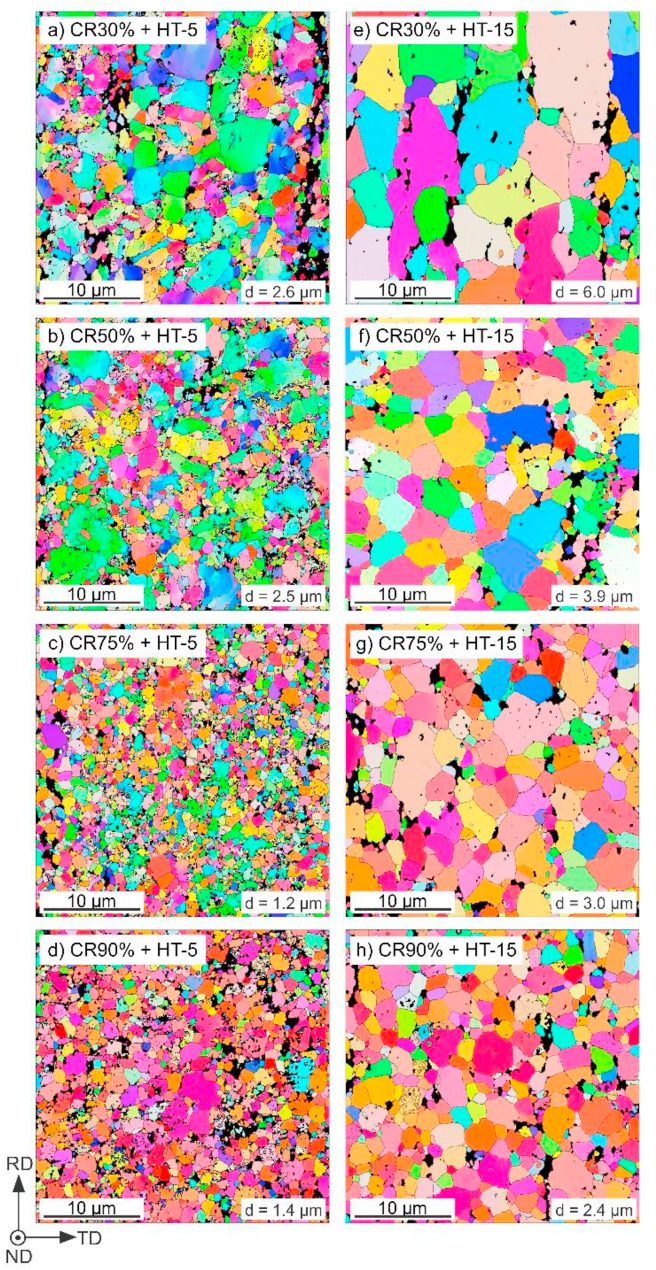
EBSD-IPF maps of Zn–3Ag-0.5Mg alloy after CR + HT-5 (a–d) and CR + HT-15 (e–h). collected in the rolling plane from 30 × 30 μm area.

**Fig. 6:**
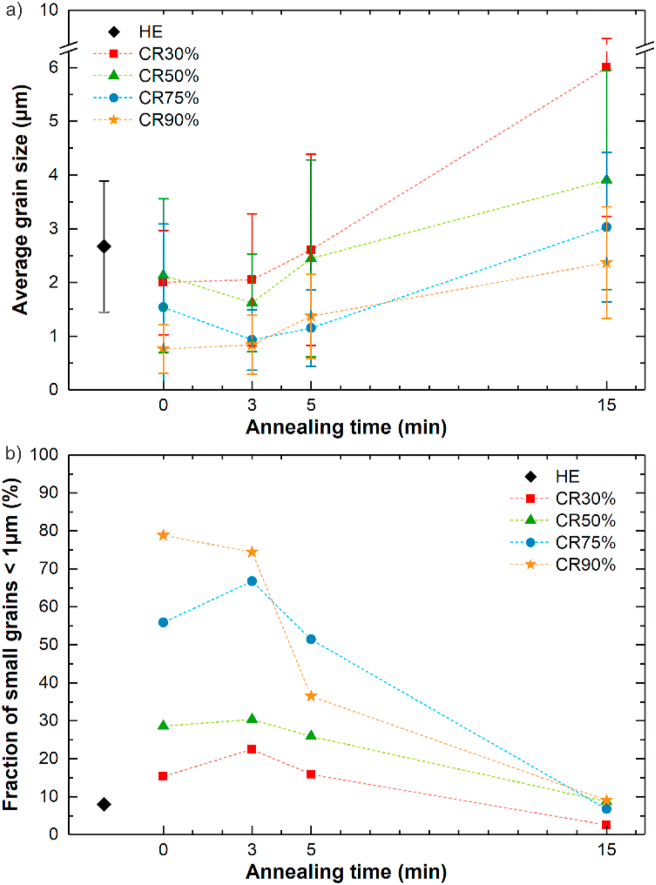
Variation of the average grain size (a) and volume fraction of small grains (b) in samples after HE and CR vs. the applied annealing time. The results present mean values of grain size and their standard deviation.

Additionally, the IPF maps of CR samples followed by HT-5 and HT-15 were shown in Fig. 5. The average grain size with standard deviation measured for all investigated samples was summarized in Fig. 6a. Also, the fraction of small grains (<1 μm) in each sample was presented in Fig. 6b.

**Fig. 7:**
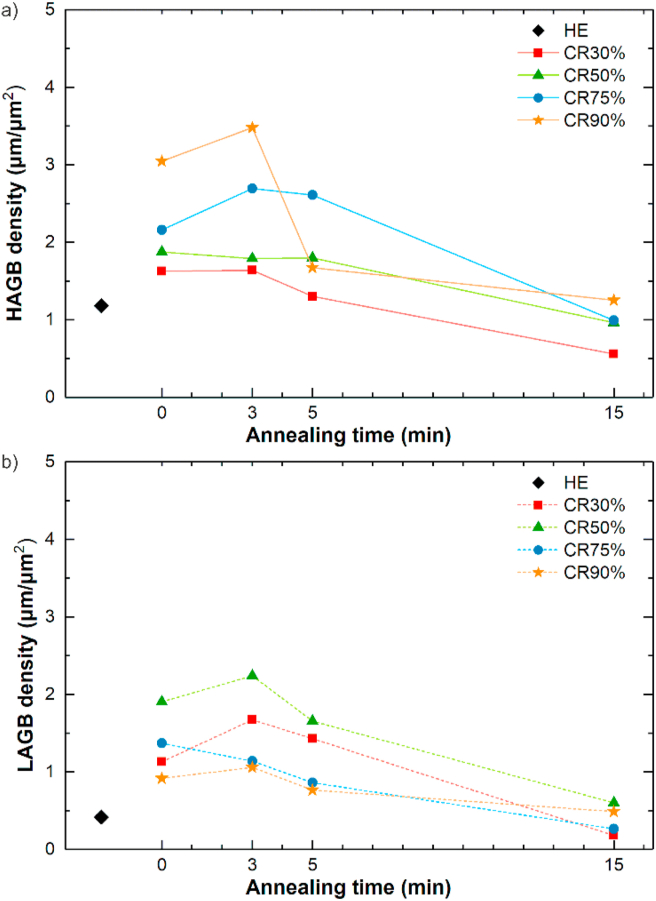
The dependence of grain boundaries density: HAGB (a), LAGB (b) in samples after HE, and CR vs. the applied annealing time.

Up to CR75%, the microstructure is composed of bigger deformed grains, with a subgrain structure, and some smaller dynamically recrystallized DRX equiaxed grains. The fraction of small grains increases with the applied CR reduction and reaches almost 80% after CR90%. The CR90% sample exhibits a homogeneous DRX ultrafine-grained microstructure. Variation of LAGB and HAGB density with increasing cold work, presented in Fig. 7 confirms these observations. The energy stored in grains deformed at higher CR reduction produces an increased number of HAGB by the occurrence of DRX and formation of dislocation-free grains with HAGB, which simultaneously limits the LAGB density.

Interestingly, the applied HT-3 resulted in a decrease in average grain size and an increase in small grain fraction in all samples, except for the CR90% state. Short-term annealing most likely gave rise to local development of polygonization processes, in which the deformation subgrain structure evolved into HAGB and resulted in the formation of small grains inside the deformed large primary grains [24].

Further annealing applied for 5 and 15 min allowed for progressive grain growth that was limited during the cold deformation due to second phases. After HT-15, HAGB and LAGB densities decreased in pursuance of the observed grain growth. The simultaneous reduction of LAGB density in the η-Zn grains is most likely caused by continuous recrystallization related to the movement of dislocations trapped in sub-boundaries with low misorientations to grain boundaries resulting in the gradual transformation to HAGB [25].

### 3.3 Texture analysis

**Fig. 8:**
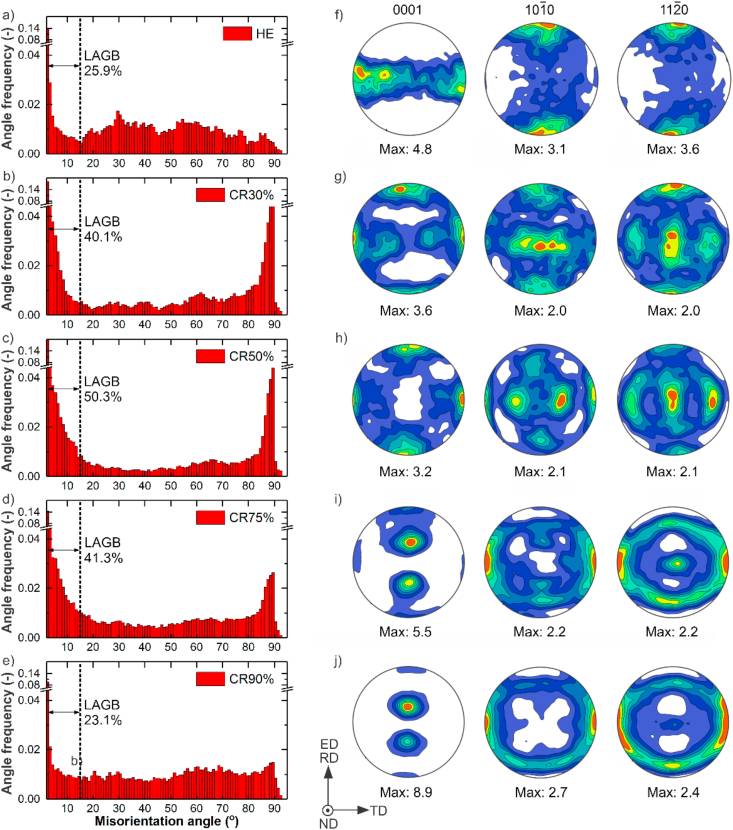
Distribution of grain boundary misorientations (a–e) and pole figures of Zn–3Ag-0.5Mg alloy in the HE (a, a1) and CR30% (b, b1), CR50% (c, c1), CR75% (d, d1), CR90% (e, e1) samples.

In Fig. 8a–e, the progressive variation of misorientation angles distributions for HE and CR states, including the change of LAGB/HAGB ratio, are shown. With an increasing CR ratio, up to 75%, the subgrain structures with LAGB develop, while at 90%, the cold work is large enough to provide almost full DRX. CR90% process produces ultrafine grains with a visible decrease in LAGB fraction in favor of the higher HAGB fraction in the microstructure compared to other CR samples. Moreover, twin boundaries formed after CR30% gradually disappeared, becoming probably the nucleation sites for newly formed grains in samples subjected to higher CR reductions.

In Fig. 8f–j basal (0001) and prismatic $\left(10\bar{1}0\right)$, $\left(11\bar{2}0\right)$ pole figures are presented. After HE, the $\left(10\bar{1}0\right)$ fiber texture is observed with all the (0001) basal plane normals perpendicular to the extrusion axis. It is typical for HCP metals with c/a ratio >1.633 [26]. In pole figures shown in Fig. 8, the evolution of texture in CR samples with increasing CR ratio is visible. The annealing did not introduce visible changes in the texture and misorientation angle distributions, therefore the results for HT samples were not included in Fig. 8.

### 3.4 Mechanical properties

**Fig. 9:**
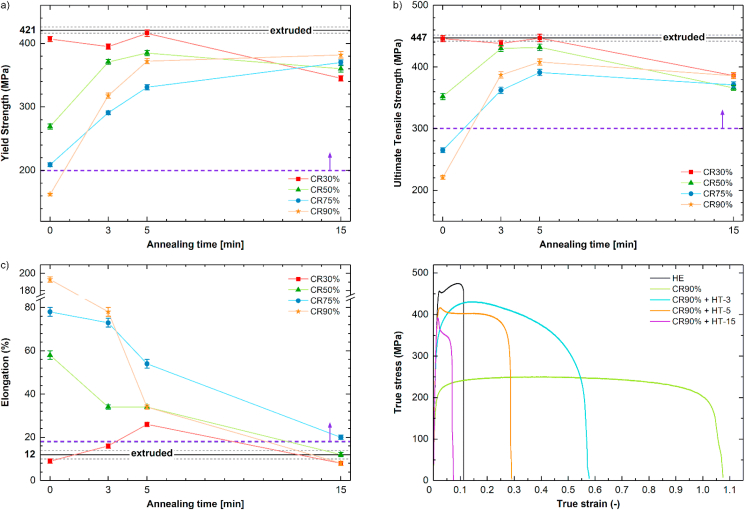
Plots of mechanical properties versus annealing time of CR samples in relation to the HE state: Yield Strength (a), Ultimate Tensile Strength (b), Elongation to failure (c), True stress-strain curves of HE and CR90% samples with applied HT (d). Dashed purple lines indicate the mechanical benchmarks for metallic biodegradable implant materials.

Mechanical properties: YS (Fig. 9a), UTS (Fig. 9b), and ε_F_ (Fig. 9c) recorded during tensile tests were presented as a function of the applied annealing time of for all CR samples. The average values measured for the HE sample were also plotted with a solid line for comparison (black dashed line indicates the standard deviation). As can be seen, the HE sample reached 421 MPa, 447 MPa, and 12% of YS, UTS, and ε_F,_ respectively. These mechanical properties perform well compared to Mg-based alloys, another group of extensively studied biodegradable materials [27]. For instance, widely used commercial Mg–Zn–Zr alloy after hot extrusion exhibits UTS, YS, and ε_F_ of 312 MPa, 378 MPa, and ~20%, respectively.

The next processing step of the Zn–3Ag-0.5Mg alloy (in Fig. 9a–c presented as annealing time = 0), led to a decrease in strength and an increase in plasticity with increasing CR reduction. Subsequent short-time annealing up to 5 min enhanced YS and UTS over 331 MPa, and 391 MPa, respectively. HT-3 and HT-5 also caused a decrease in plasticity, however, total ε_F_ was still much over the marked limit of 18% elongation for biodegradable implant materials. Further annealing for 15 min is no longer favorable for the plasticity and UTS, as both properties decreased.

In the CR30% sample, a slightly different tendency in mechanical behavior variations was observed. Up to 5 min of annealing, both YS and UTS were similar to the values recorded for the HE sample and decreased only after a longer time of HT. In contrast to the samples deformed with higher CR reductions, elongation initially slightly increased and then decreased below 12% after applied HT-15. This may be due to the relatively small CR reduction, so the presence of HE microstructure residues in the microstructure and, at the same time, significant texture changes.

Based on the results presented in Fig. 9, the CR process followed by 5 min annealing gives optimal mechanical properties. Interestingly, CR50% and CR90% samples exhibit the most promising mechanical behavior: UTS of 432 MPa and 408 MPa, YS of 385 MPa and 372 MPa, respectively, and ε_F_ of ~34% for both materials. For comparison, the mechanical properties achieved in the Zn–5Ag-0.5 =Mg alloy processed by unidirectional hot rolling with a cumulative reduction of 80% are YS = 258 MPa, UTS = 356 MPa, and ε_F_ = 42%. It proves that the combination of cold rolling with appropriate short-term HT used in our studies HT gives favorable mechanical results [28].

True stress-strain curves are shown for the CR90% sample in Fig. 9d to present the deformation behavior during tensile tests. Because of significant differences in the deformation behavior of samples subjected to tensile tests, lack of evident yield point, and a nonlinear elastic region observed in some of the investigated samples; a proof YS was carefully estimated by using a higher strain offset up to 0.5%. This approach has been applied in other hcp alloys and provided better reproducibility of YS measurements and more reasonable results [29,30]**.** CR, followed by HT, clearly affects the deformation mechanisms activated during straining compared to the HE sample. After exceeding the flow stress peak, CR samples undergo marked strain-softening leading to large elongation, especially ε_F_ in CR90% sample reached almost 200%. A similar phenomenon was observed in Zn–4Ag and Zn–4Ag-0.6Mg alloys with highly refined microstructures after cold drawing [15]. The observed strain-softening is attributed to occurring DRX after reaching the peak flow stress, and GBS activated in ultrafine-grained Zn alloys [11].

**Fig. 10:**
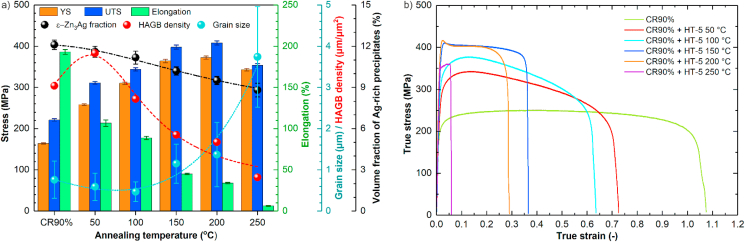
Tensile test results, average grain size, HAGB density, and ε-Zn_3_Ag precipitates' volume fraction for the CR90% samples subjected to HT-5 at different annealing temperatures (a), True stress-strain curves of CR90% + HT-5 at different annealing temperatures (b).

In addition to the presented results, the effect of annealing temperature on the grain size, HAGB density, and ε-Zn_3_Ag precipitates' volume fraction on the CR90% sample's mechanical properties annealed for 5 min was presented in Fig. 10. The increase in annealing temperature resulted in a slight gradual grain refinement up to 100 °C and, above this temperature, a marked grain growth. In general, the average grain size increased from 0.59 μm in CR90% + HT-5 50 °C up to 3.74 μm in the HT-5 250 °C sample. A successive decrease in HAGB density accompanied changes in an average grain size value. When analyzing the correlated results of grain size and HAGB density with mechanical properties, similar relations between these parameters and different applied annealing times can be noticed. Besides, the ε-Zn_3_Ag precipitates' volume fraction decreased from ~12% after CR90% to ~9% after the CR90% + HT-5 250 °C. Simultaneously, the amount of Mg-rich grains remained unaffected within this temperature range.

### 3.5 Vickers microhardness

**Fig. 11:**
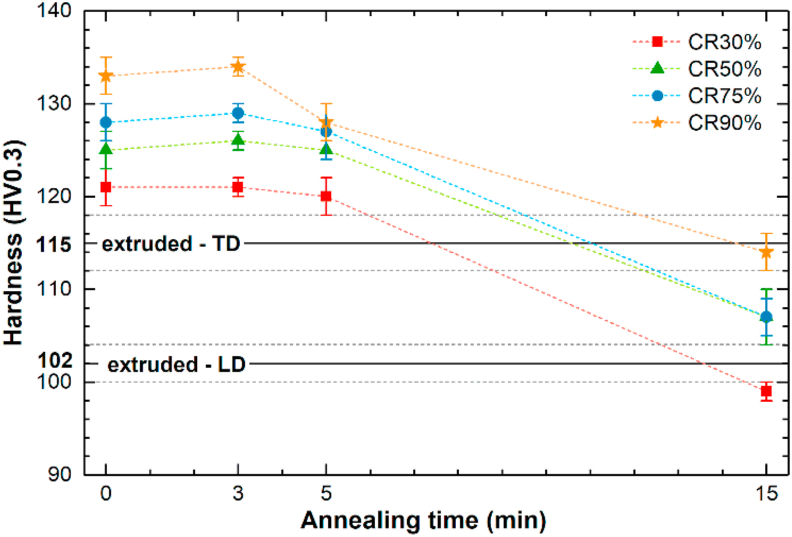
Average microhardness results for the samples in the HE, CR, and CR + HT states. (microhardness of the HE sample measured in two perpendicular directions, on the longitudinal – LD, and the transverse cross-sections - TD).

Fig. 11 shows the changes in average Vickers microhardness for the CR samples as a function of annealing time. Additionally, the microhardness of the HE sample was measured on transverse (TD) and longitudinal (LD) cross-sections to check the texture effect, and the average results with standard deviation are plotted in Fig. 11.

It can be seen that CR samples exhibit significantly higher microhardness than the HE samples in both directions. With an increasing CR ratio, an increase in microhardness, up to 133 HV0.3 for the CR90% sample, is noticeable. After HT-3, the average microhardness did not show a significant change. After HT-5, the average microhardness slightly dropped. While HT-15 resulted in an evident decrease in microhardness accompanied by the observed grain growth. The CR30% + HT-15 sample with a grain size of ~6.0 μm showed the largest decrease in microhardness by 22 HV0.3 compared to the CR30% state. Besides, the highest microhardness in the HT-15 state is represented by the CR90% sample and is close to the microhardness of the Zn–3Ag-0.5Mg alloy achieved in the HE state measured on the TD cross-section.

## 4 Discussion

### 4.1 Hot-extruded state

In the current study, HE process applied directly to the as-cast Zn–3Ag-0.5Mg alloy provided finer microstructure (grain size of ~2.7 μm) than in the previously reported Zn–3Ag-0.5Mg HE sample processed with inter-process annealing (grain size of ~6.0 μm). Grain refinement and crushing fine eutectic net resulted in substantially improved plasticity (ε_F_ increased from ~4% to 12%) and mechanical strength higher by ~49%, ~25% in the case of YS and UTS, respectively. It has already been found that the Zn-0.8Mg-0.2Ca alloy fabricated by extrusion of non-annealed initial material reaches higher mechanical properties due to the smaller size of η-Zn grains and the Mg_2_Zn_11_ intermetallic precipitates [31]. This indicates that the omission of HT before the plastic deformation process for certain Zn alloys exhibiting uniform microstructure after casting can be beneficial for their mechanical parameters.

As seen from Fig. 10, the investigated Zn–3Ag-0.5Mg alloy in the HE state possesses the highest YS and UTS among the tested samples. Such high strength is attributed to the microstructure composed of grains possessing a strong basal fiber texture oriented so that basal planes are parallel to the tensile direction. Under loading in the direction parallel to ED, the basal slip system fails to accommodate the deformation, as the Schmid factor is close to zero for this slip system. Since the easy slip system is effectively blocked, grains are more susceptible to twinning or non-basal slip at the beginning of deformation [32]. The indirect evidence for twinning activation during HE sample deformation is a peak in misorientation angle distribution related to $\left(10\bar{1}2\right)\left[10\bar{1}1\right]$ twin boundary at ~86° observed in CR samples. Typically, when the contribution of twinning in the total deformation of Zn-based alloys increases, a higher strengthening effect, and lower plasticity is observed [6,9,17]. Therefore, despite the excellent tensile strength of the Zn–3Ag-0.5Mg alloy, inadequate ductility in the HE state was still an issue to address in further processing steps.

Vickers microhardness measured in the HE state in TD and LD cross-sections reached 115 HV0.3 and 102 HV0.3, respectively. The microstructure after HE can be described as fully recrystallized and equiaxed. Therefore, the hardness anisotropy is most likely related to the crystallographic texture. It was shown for Zn-based thin coatings that the microhardness measured in the *a*-axis is over 2.5 higher than in *c*-axis [33]. Accordingly, in this current study, in the TD cross-section of the Zn–3Ag-0.5Mg HE sample, most of the grains have a *c*-axis oriented perpendicular to the loading direction, resulting in higher resistance to the applied load and, thus, higher microhardness. The fiber texture results in grains having a *c*-axis aligned at an angle range between 0° and 90° to the loading direction in the longitudinal cross-section. This wide distribution of *c*-axis orientations in the longitudinal cross-section results in easier accommodation of the deformation by the active slip systems, and therefore lower measured microhardness.

### 4.2 Effect of cold rolling on the microstructure and mechanical properties

Microstructural and mechanical investigations of the multi-phase Zn–3Ag-0.5Mg alloy revealed that even subtle changes in grain size, texture, fraction, and type of grain boundaries and the intermetallic precipitates' volume fraction markedly affect the mechanical behavior of the Zn alloy.

It was shown that hard precipitates of second phases or ceramic particles in Mg or Zn alloys could promote the DRX process via the particle stimulated nucleation mechanism [19,34]. Thus, the presence of Ag- and Mg-rich precipitates in the investigated Zn–3Ag-0.5Mg alloy might contribute to DRX and average grain size reduction. The formation of recrystallized grains occurred in places of localized strain, such as grains adjacent to the second phases' precipitates [25]. DRX was likely to occur at RT in this case due to the low recrystallization temperature of pure Zn, resulting from its low melting temperature (T_m(Zn)_) of 419.5 °C. It also means that maintaining the highly deformed microstructure in Zn-based materials with a high density of dislocations and defects inside the deformed grains is not easy since they are immediately annihilated at RT recovery and recrystallization processes [35]. Moreover, it cannot be ignored that even at relatively slow rolling speeds, the internal friction of the processed material or the frictional heat generated between the rolls and strip may slightly increase the temperature of the process above RT and soften the material between the CR steps due to the dynamic recovery (DR) and/or DRX processes. As shown in our previous studies, CR75% produced a grain size of ~1.8 μm, while high-pressure torsion for 5 turns refined grain size only to ~1.2 μm [21]. It is apparent that even without high accumulative strain, it is possible to activate DRX in this material. Moreover, similar results were observed in Zn-0.34Mn and Zn-0.76Mn alloys after cold rolling with a total reduction of 84% [36]. Therefore, even after low CR reductions, the grain refinement started to occur, like in the CR30% sample.

The pronounced grain refinement is related to an increase in the number of new refined DRX grains with size <1 μm (Fig. 6). According to the Hall-Petch relationship concerning grain boundary strengthening, there should be an inverse dependence of hardening ability on the grain size [37]. However, as observed in the current study, reducing the grain size in Zn–3Ag-0.5Mg alloy below ~2 μm delivered an opposite result. Grain refinement led to the desired plasticity enhancement, however, it simultaneously led to decreased yield and flow stresses with increasing CR reduction. This can be attributed to the inhibition of twinning in samples with ultrafine grain size in favor of other deformation mechanisms [38]. The low melting temperature of Zn allows for high-temperature deformation systems, such as prismatic and pyramidal slip systems, GBS, or diffusional creep to be activated at RT that represents a relatively high homologous temperature (~0.43 T_m(Zn)_) [[39], [40], [41]]. According to our previous findings, this change in the dominant deformation mechanism from twinning to slip or GBS contributes to evident strength decrease [11,21]. The subgrain structure consisting of a relatively high density of LAGB, developed during CR processing, does not provide an effective barrier for dislocation slip. However, GBS is known to be misorientation angle-dependent, and in Zn-based materials, it is easier at HAGB than LAGB [42,43]. Thus, a large amount of HAGB in the CR90% sample provided the best platform for GBS sliding activation during tensile deformation, resulting in an ε_F_ of almost 200%, YS of 162 MPa, and UTS of 221 MPa.

Moreover, the observed decrease in strength compared to the HE state can be related to precipitation-induced softening. It has already been presented for the Zn–1Cu alloy that extensive cold deformation processing can contribute to the precipitation of fine secondary phases from non-equilibrium η-Zn(Cu) solid solution [44]. A similar situation may occur in the examined CR Zn–3Ag-0.5Mg alloy since the matrix is composed of η-Zn(Ag) solid solution, and similar precipitations of the ε-Zn_3_Ag intermetallic phase may occur. This effect seems to be more pronounced in samples deformed with higher CR reductions. A high fraction of deformation-induced secondary precipitates could affect the observed mechanical properties by activating phase boundary sliding (PBS) at the matrix-precipitate interphase boundaries instead of dislocation – precipitate interaction [15,44].

The relevant influence of microstructure on mechanical properties can also be observed when comparing the results for the Zn–3Ag-0.5Mg alloy after CR75% to the same CR reduction in our previous studies but preceded by inter-process annealing [21]. Simultaneous plasticity improvement (ε_F_ increased from 53% to 78%) and decreased mechanical strength (a decrease in YS and UTS from 254 MPa to 209 MPa and from 382 MPa to 265 MPa, respectively) occurs in the samples extruded straight after casting. It is accompanied by a decrease in grain size from ~1.8 μm to ~1.5 μm and an increase in HAGB fraction from ~47% to ~63%. Thus, it is more likely that GBS occurs to a greater extent in place of twinning or slip during tensile deformation, which softens the investigated here material. Also, a significantly smaller size of Mg-rich grains formed because of crushing the eutectic net during plastic deformation of the as-cast material may be the reason for DRX-enhanced better plasticity.

Interestingly, in contrast to the decrease in YS and UTS in CR samples, the Vickers microhardness improved with an increasing CR ratio, up to 133 HV0.3 in the CR90% sample. This strengthening effect could be affected by different grain orientations related to the applied load. For instance, a strong texture with regard to the applied load during the tension of the HE sample is relatively weak for the deformation in the perpendicular direction induced by microhardness indentation. In CR samples, an increasing applied total strain leads to the formation of a typical rolling texture with two (0001) poles tilted ~15–25° away from ND toward the RD (Fig. 8d1). The prismatic $\left(10\bar{1}0\right)$ and $\left(11\bar{2}0\right)$ poles are more axisymetrically distributed (in respect to ND) with a tendency to concentrate around TD, similar to Zn–Cu–Ti rolled sheet presented in Ref. [45]. Nevertheless, in terms of microhardness, although the orientation of grains in CR samples was gradually changing towards the “soft” *c* direction, the grain size was decreasing and, together with precipitation hardening by a higher fraction of uniformly distributed deformation-induced second phases' precipitates, it seems to have a greater influence on the higher microhardness. Despite the low applied load, the deformed area and indent size during microhardness tests were one order of magnitude larger than the average grain size. Therefore, it can be assumed that the measurements were more sensitive to the number of grain boundaries and grain size than the orientation of a single grain, especially when the grains in the CR samples presented a certain orientation distribution within the microstructure instead of a strong texture.

The opposite effect can also be related to the tension-compression asymmetry and anisotropy of the HCP crystal structure influencing the mechanical properties variation. It is worth mentioning that, according to our previous research, GBS occurs much easier under tension than compression [46]. Knowing that GBS is a dominant mechanism activated during the deformation of ultrafine-grained CR samples, the differences in CR samples' strengthening effects can be explained by the contribution of GBS in total deformation. Assuming that microhardness is measured at a much higher strain rate than in static tensile testing, the mechanism of GBS occurring during deformation in ultrafine-grained materials is suppressed in microhardness measurements and has a lower impact on the strain-softening observed in tensile testing. As a result, a decrease in yield and tensile strength with the decreasing grain size simultaneously contribute to microhardness enhancement.

Finally, the relatively large volume fraction of second phases' precipitates in samples after deformation with higher CR reductions might lead to a decrease in strength by GBS, but simultaneously increase the microhardness by precipitation hardening effect. It was shown by Li et al. that in the Zn–4Ag alloy, precipitation hardening slightly increases the hardness, whilst YS and UTS decrease [47].

Importantly, CR processing with a 90% reduction of the Zn–3Ag-0.5Mg alloy results in one of the highest microhardness (133 HV0.3) among the other biodegradable multi-phase Zn alloys after plastic deformation, as documented so far [48].

### 4.3 Effect of short-time annealing on the microstructure evolution and mechanical properties

#### 4.3.1 Effect of 3-min annealing on microstructure and mechanical properties

Short-time HT subsequently applied after CR allowed for strength restoration and the simultaneous limitation of plasticity. In order to analyze the effect of applied short-time HT-3 annealing, the CR samples were divided into two groups: 1) deformed, partially recrystallized CR30%, CR50%, CR75% samples; 2) almost entirely recrystallized CR90% sample. As seen in Fig. 6b, the fraction of small grains in the microstructure of samples from group 1 increased compared to the CR state. The substructure observed during EBSD analysis of bigger deformed grains after CR presumably transformed into new grains with HAGB due to the polygonization process. This may explain maintaining the grain size in the CR30% sample at the same level of ~2 μm, and the unexpected grain size reduction in CR50% and CR75% samples by ~24% and ~40%, respectively, after the HT-3. LAGB and HAGB density stayed almost unchanged or slightly increased in these samples. The increased HAGB density in the CR90% + HT-3 sample is accompanied by the decrease in small grains fraction and a slight increase in average grain size. It may result from the growth of small DRX grains with HAGB and the formation of new HAGB in places with any residual energy stored in dislocations after deformation considered as LAGB.

Interestingly, during annealing at 200 °C (~0.68 T_m(Zn)_) the volume fraction of ε-Zn_3_Ag phase markedly increased compared to the CR state, up to CR75% reduction. A similar phenomenon has already been observed in the CR WE43 Mg alloy, where in the first stage of short-time annealing (at 400 °C, ~0.72 T_m(Mg)_), the precipitation of small intermetallic particles occurred concurrently to the recrystallization process along deformation twin boundaries and grain boundaries formed within the matrix as a result of applied cold work. Longer annealing resulted in the coarsening of those precipitates and, finally, the expected dissolution of the majority of precipitates occurring in the microstructure. This unusual observation was explained by the combined effect of solute segregation near twin and grain boundaries and high local residual strain [49]. This could be an explanation for the increased amount of ε-Zn_3_Ag in the CR + HT-3 Zn–3Ag-0.5Mg alloy's microstructures. Also, the 3-min annealing might be too short for Ag atoms diffusion and, consequently, dissolution of Ag-rich particles in the matrix. Therefore, it is supposed that HT-3 annealing acts like accelerated aging and leads to precipitation of ε-Zn_3_Ag particles from the non-equilibrium matrix remained after CR. In contrast, the ε-Zn_3_Ag volume fraction decreased by ~18% in the CR90% sample.

As the activation of GBS, which provides remarkable plasticity in Zn alloys, depends on the grain size and is easier to activate in ultrafine-grained materials, an evident correlation between the small grains volume fraction and recorded ε_F_ can be found. In CR90% and CR75% samples subjected to HT-3, the ε_F_ was measured to be above 70% with the fraction of small grains equal to ~74% and ~67%, respectively, and with the average grain size below 0.9 μm in both cases. Nevertheless, it should be emphasized that the decrease in ε_F_ from 193% to 78% in the CR90% sample, as an outcome of HT-3, resulted not only from a small change in average grain size from ~0.76 μm to ~0.84 μm, but had to be accompanied by the decreased amount of ε-Zn_3_Ag precipitation-matrix interphase boundaries, limiting the PBS.

The observed microstructural changes after HT-3 did not significantly affect the Vickers microhardness of CR samples.

#### 4.3.2 Effect of 5-min and 15-min annealing on microstructure evolution and mechanical properties

Further 5-min and 15-min annealing caused a progressive grain growth, visible decrease in small grains volume fraction, and a decrease in grain boundary densities. A clear relationship between grain size, HAGB density, and elongation can be drawn. For instance, the increase in grain size by ~60% and the decrease in HAGB density by ~50% in the CR90% + HT-5 sample resulted in the decrease in ε_F_ from 78% to 34% compared to the HT-3 state. The observed grain growth could limit the GBS during tensile deformation in favor of dislocation slip and restore the twinning as one of the dominant deformation mechanisms. It might explain the evident increase in YS values and decrease in plasticity due to the twinning-related η-Zn(Ag) matrix strengthening and accelerated fracture after HT-15 before reaching elongation of 20%.

Moreover, the dissolution of Ag-rich precipitates observed as a decrease in precipitates' volume fraction after HT-15 is equivalent to the enrichment of the η-Zn(Ag) matrix in Ag atoms, and therefore, a larger solid-solution strengthening effect and higher YS [50]. Interestingly, UTS achieved higher values after HT-3 and HT-5, which suggests that strain-softening was suppressed to some extent, but applied HT-15 caused the decrease in flow stresses. Nevertheless, a similar observation was reported for the Zn–1Cu alloy, where the gradual precipitates' dissolution and grain growth during short-time annealing resulted in the decrease in UTS during tensile tests [44].

Enabling strain hardening by ensuring adequate YS and relatively high UTS is highly desirable for considered structural Zn alloys' medical applications. For instance, (1) the high strain hardening rate may contribute to an increase in strength during stent expansion; (2) high UTS may deliver appropriate radial strength and limit the diameter of struts used for stent production; (3) satisfactory ductility may provide the stent to withstand deformation during expansion without fracture [51]. The average calculated UTS/YS ratio was close to ~1.3 for CR samples and gradually decreased from ~1.2, to 1.1 to almost ~1.0 for HT-3, HT-5, and HT-15, respectively. A decreasing tendency of strain hardening was accompanied by the CR and CR + HT samples' grain growth and a smaller amount of Ag-rich hard precipitates what reduced the number of obstacles for impeding dislocations movement. This indicates the smaller strain hardening ability obtained in CR + HT-15 samples with the biggest grain size and the smallest Ag-rich precipitates' volume fraction.

In all CR samples, the HT resulted in a decrease in microhardness, to a small extent after HT-5 and significantly after HT-15, which was accompanied by observed grain growth. Furthermore, after HT-15 in CR samples, the microhardness dependence was mostly related to the grain size, as there was no significant difference in the grain orientations, as seen in the IPF maps (Fig. 5). After HT-15, the highest microhardness of 114 HV0.3 was measured for the CR90% + HT-15 sample with the average grain size of ~2.4 μm. This is close to the value measured in the HE sample in TD, with the grains mostly in the ‘hard' $\left[11\bar{2}0\right]$ orientation. Similar behavior was observed in the Zn–Al and Zn–Al–Cu alloys subjected to CR90% reduction and subsequent annealing at 250 °C for 1–1440 min [52]. At the beginning, the increase in annealing time results in grain coarsening and hardness enhancement (the inverse Hall-Petch relation), but when the grain size exceeds the critical value of 1.8 μm and 0.8 μm in Zn–Al and Zn–Al–Cu alloys, respectively, a typical annealing-softening behavior appears, and a decrease in hardness takes place. The grain refinement below the critical size results in a higher density of HAGB, sensitive to dynamic recovery, leading to the annihilation of dislocations during deformation and thus, softening the ultrafine-grained material. A decrease in Ag-rich precipitates' volume fraction and simultaneous enrichment of the matrix in Ag atoms does not seem to enhance the microhardness by solid-solution hardening.

It is also worth pointing out that compared to the mechanical properties of samples in the CR + HT-3 state, the HT-5 annealing resulted in a decrease in plasticity, an increase in strength (both YS and UTS), and also the unusual slight decrease in microhardness. This result can be analogously explained as it was done for CR samples with different total strains. An increase in average grain size, a decrease in HAGB, and the fraction of small grains hinder GBS responsible for strain-softening of the deformed samples. As mentioned, the GBS has a much bigger significance during tension than compression, so the disappearance of GBS does not give rise to measured microhardness as it can be observed for tensile strength. Moreover, with the increasing annealing time, Ag-rich particles' dissolution was noted and may impede the effective precipitation hardening relevant to microhardness. Considering a decrease in microhardness with a grain growth, the Hall-Petch relation related to the grain-boundary strengthening seems preserved in this case and continues to maintain in the context of microhardness at longer HT-15 annealing.

### 4.4 Effect of annealing temperature on the mechanical properties of the CR90% sample

To the best of the authors' knowledge, the influence of annealing temperature after plastic deformation on the microstructure and mechanical properties has never been investigated for high-strength Zn alloys considered for biomedical applications. The plastic deformation process is an integral part of implant fabrication. Knowing the possible benefits of mechanical performance improvement by applying short-time heat treatment at relatively low temperatures as an additional, low-cost processing step can be highly valuable.

It was found that the main changes in the microstructure of the CR90% sample after HT-5 annealing at different temperatures are related to the grain size, HAGB density, small matrix grains, and ε-Zn_3_Ag precipitates volume fraction without relevant texture changes. That is why the relations between mechanical properties and analyzed microstructural features seem consistent with already discussed relations in samples after different CR reductions and different used HT times. The microstructural parameters and tensile properties collected in one graph (Fig. 10) clearly show their interactions. It is challenging to distinguish simple relation between a single microstructural parameter and strength or elongation to failure. Thus, the analysis must be approached comprehensively.

In typical metallic materials, the applied heat treatment after the cold deformation process conducted at different annealing temperatures allows for determining the conditions necessary for full recrystallization of the deformed microstructure and subsequent grain growth [25]. However, as it has been already mentioned, any plastic deformation applied to Zn alloys results mainly in the DRX process, since maintaining the energy in the deformed Zn grains is hardly possible, and only some residues of dislocations can remain after cold plastic deformation processes. This explains a slight decrease in grain size after low-temperature annealing (<100 °C) related to possible polygonization in the microstructure and forming a new fraction of small grains due to LAGB to HAGB transformation. In general, with gradual grain growth and a further decrease in HAGB density at higher annealing temperatures, the ductility deteriorates. Change of the main deformation mechanism from GBS and extensive DRX during the tensile test after reaching the flow point to dislocation slip and after HT-5 250 °C also twinning visible in true stress-strain curves is responsible for the observed improvement of mechanical strength in HT Zn–3Ag-0.5Mg alloy. In particular it enhanced UTS and YS, reaching the maximum value for the HT-5 200 °C sample with the following mechanical properties: UTS of 408 MPa, YS of 372 MPa, which compared to the CR90% state gave rise by 85% and 130%, respectively. Importantly, with the obtained high mechanical strength, the elongation remained at a satisfactory level of 34%. The dissolution of ε-Zn_3_Ag precipitates with increasing annealing time also influences the mechanical properties by strengthening the η-Zn(Ag) matrix solid solution and decrease in interphase matrix-precipitate boundaries providing an easy way for sliding.

### 4.5 The explanation for the microstructure-dependent fine-tuning of mechanical properties in Zn alloy

Comprehensive analysis of the effect of HE, CR, and HT processes on Zn–3Ag-0.5Mg alloy's tensile properties provided the following insights. (1) Relatively large DRX grains formed in the Zn–3Ag-0.5Mg alloy after HE, having a basal fiber texture in tensile/extrusion direction, are prone to twinning and further dislocation slip during tensile testing. This is accompanied by significant work hardening up to the maximum value of stress concentration caused by the interaction of deformation twins and slip bands, resulting in an abrupt fracture without necking. (2) According to CR samples' total elongation obtained in tensile tests, they can be assigned to two groups: exhibiting a moderate elongation after CR30% and CR50% thickness reduction and large elongation above CR75%. In the first group, dislocation slip-induced strain hardening is assumed to happen first, and then DRX is activated since the decrease in stress is observed during continuous tensile deformation. The microstructure in those samples containing both smaller DRX grains, and larger ones with subgrain structure evolving from LAGBs to HAGBs during deformation, allows for some strain hardening resulting in high tensile strength and acceptable plasticity. The second group of samples subjected to large total deformation have the average size of equiaxed grains smaller than 1.5 μm. They are almost entirely DRX in the whole volume of the sample. A high fraction of HAGB and increased matrix – ε-Zn_3_Ag precipitation interphase boundaries cause an easy activation of GBS and, as a result, decreased strength and excellent plasticity. After CR75% and CR90%, the fraction of HAGB becomes sufficient to obtain microstructure resistant to further grain growth. (3) Short-time HT conducted at lower annealing temperature applied to CR90% sample results in improved mechanical strength (compared to ultrafine-grained samples after CR90%) by a slight grain growth and limitation of GBS and DRX in favor of dislocation slip. The continued increase in UTS at higher annealing temperature results from even larger grains allowing for strain hardening by twinning.

The presented results suggest that grain refinement down to an ultrafine level does not provide successful mechanical strength enhancement in Zn–3Ag-0.5Mg alloy, as it is typically known from other metals [53]. Nevertheless, the introduction of alloying elements providing solid-solution strengthening and second phase strengthening can provide a proper platform for the thermo-mechanical strengthening of Zn alloys. Based on current studies, it is highly advisable to apply the cold deformation process to high strength, brittle multi-phase Zn alloys to provide adequate plasticity by grain refinement at first, and then to employ short-time annealing treatment to restore proper strength. In short, the adequate combination of CR reduction and subsequent HT provided a meaningful plasticity improvement in the high-strength brittle HE Zn–3Ag-0.5Mg alloy.

The joint effect of grain refinement strengthening, second phases precipitation hardening, favorable texture, and an appropriate fraction of LAGB and HAGB can provide mechanical properties meeting the requirements for biodegradable implant materials.

## 5 Conclusions

The effect of cold rolling and subsequent short-time annealing on the hot-extruded high-strength Zn–3Ag-0.5Mg alloy was investigated through microstructure, texture, and mechanical properties analysis.

1. With increasing CR ratio, a fine-grained microstructure with the smallest grain size of ~0.76 μm, a typical rolling texture, and the highest fraction of deformation-induced precipitates of the ε-Zn_3_Ag phase, was obtained after 90% of thickness reduction. Short-time annealing resulted in a gradual increase in grain size of up to ~6.0 μm in the CR30% + HT-15 sample, with almost no texture changes.
2. Fine-tuning of mechanical properties in the novel Zn–3Ag-0.5Mg alloy was possible by the cold plastic deformation process and post-deformation annealing. The applied processing route allowed for controlling the grain size, texture, fraction of second phase precipitations, and HAGB/LAGB densities and resulted in an excellent combination of strength and plasticity in CR Zn–3Ag-0.5Mg alloy.
3. The analysis of the applied annealing temperature-dependent HT shows that ultrafine-grained material obtained after CR90% exhibiting properties of high ε_F_ of ~200%, but insufficient mechanical strength (YS = 162 MPa and UTS = 221 MPa) can be easily enhanced up to the required values by short-time post-deformation annealing already at 50 °C. However, the peak for mechanical strength (YS = 372 MPa and UTS = 408 MPa) and optimal ductility (ε_F_ = 34%) was found at 200 °C.
4. The most promising properties obtained in the CR50% + HT-5 sample, such as UTS of 432 MPa, YS of 385 MPa, and ε_F_ of 34%_,_ clearly exceed the mechanical benchmarks for biodegradable implant materials, e.g. cardiovascular stents, and makes this material valuable for further research related to biodegradation behavior and biological aspects crucial for possible candidates for biomedical applications.

Nevertheless, the limitation due to the strain-softening occurring by activation of grain and phase boundary sliding of Zn alloys still remains a matter of concern, requiring further mechanical research, as it can lead to localized deformation and a possibility of premature failure of the implant during operation in the human body. Moreover, the stability of the microstructure, after CR and subsequent HT and thus mechanical properties should also be taken under further consideration.

## CRediT authorship contribution statement

**Maria Wątroba:** Conceptualization, Investigation, Methodology, Formal analysis, Data curation, Visualization, Writing – original draft, Funding acquisition, Project administration. **Wiktor Bednarczyk:** Investigation, Methodology, Writing – review & editing. **Jakub Kawałko:** Formal analysis, Software, Writing – review & editing. **Piotr Bała:** Resources, Supervision, Writing – review & editing.

## Declaration of competing interest

The authors declare that they have no known competing financial interests or personal relationships that could have appeared to influence the work reported in this paper.
